# Ernest Hart: Editor of the British Medical Journal 1866–1898

**DOI:** 10.1177/09677720221135122

**Published:** 2022-11-24

**Authors:** Kenneth Collins

**Affiliations:** Medical History, 3526 University of Glasgow, Glasgow, UK

**Keywords:** *British Medical Journal*: social reform, public health, aconite poisoning

## Abstract

Ernest Abraham Hart (26 June 1835–7 January 1898) was the long-time editor of the British Medical Journal. He held strong opinions, and was often controversial but his views generally prevailed. He was born into a Jewish family in London and was educated at the City of London School. He studied medicine at the St George’s Hospital School of Medicine and specialised in diseases of the eye. His medical journalism began with The Lancet in 1857 and in August 1866, he was appointed editor of the British Medical Journal taking it, in his decades of leadership, from a small publication to a significant scientific journal increasing the British Medical Association membership substantially. Julia Frankau's novel of scandal, Dr Phillips: A Maida Vale Idyll (1887) published under the pseudonym of Frank Danby, has a leading character, Dr Phillips, thought to be modelled on Ernest Hart and who murders his wife reviving speculation about the death of Hart's first wife from accidental poisoning.

Hart was born in London, the son of a Jewish dentist He attended the City of London School, where he was an outstanding pupil gaining numerous school prizes, attaining the school captaincy, and in competition with John Seeley, the Lambert Jones scholarship. This scholarship entitled the holder to a place at the Queens’ College, Cambridge, but, as a Jew, and therefore subject to the University Test Acts, which confined admission to Anglicans, Hart could not take up the place. He studied medicine at the St George's Hospital School of Medicine, which was formally opened in 1835 and affiliated with the University of London, soon after the latter's establishment, in 1836. As a student during the Crimean War, he took a leading role in the founding of a society for medical students which campaigned for better conditions for the Officers of the Naval Medical Service.

## Medical career

Hart began his medical career working with William Coulson in Frederick Place, Old Jewry. For 2 years, he was a surgical registrar and demonstrator of anatomy at the St George's Hospital School of Medicine. In February 1859, he was appointed junior surgeon at the West London Hospital, and he became a full surgeon in September 1860 before resigning in 1863. In 1861, Hart played an important part in founding the Medical Society of London, a body that encouraged the discussion of medical subjects by students and the newly qualified. In the same year he returned to St Mary's, where he became a successful ophthalmic surgeon (1861–1868), aural surgeon (1865–1868) and Dean of the Medical School (1863–1868). During this period, he contributed various practical papers to the transactions of the Royal Medical and Chirurgical Society and to the reports of the Moorfields Ophthalmic Hospital.

During his time as a practising surgeon, Hart introduced a new technique for treating an aneurysm of the popliteal artery. However, his main specialisation was as an ophthalmologist, developing a successful practice that gave him a high income from early in his career, earning an average of over £2000 per annum during his first 5 years of practice. This gave him the means for his philanthropic activities with involvement in many medical and welfare organisations and providing funds for medical research and the scholarships to support it.

## Death of Hart's first wife

Hart's first wife, Rosetta Levy, died in November 1861 following the night-time accidental ingestion of aconite tincture instead of her intended medication, known as the ‘black draught’ which included senna, ginger, liquorice and cardamon.^1^ The aconite tincture, prepared from monkshood or wolfbane, is highly toxic but in traditional medicine extracts of Aconitum, species have been given orally as a sedative to reduce fever, to relieve pain and inflammation, for its diuretic properties and to slow heart rate but death from cardiac arrhythmias is not uncommon. Accidental poisonings are not uncommon even today.^2^ Hart was worried by his wife's vomiting, but she seemed fine the next morning when she was seen by her own doctor. Yet, 2 h later, she was dead despite the administration of three doses of dilute hydrocyanic (prussic) acid used by Victorians to control dyspepsia and vomiting. At a hearing of the Coroner's Court, the verdict was of death by accidental poisoning with the tincture of aconite. Though it was the name of Thomas Wakley, Hart's former employer and colleague at The *Lancet* that was on the death certificate was not presiding at the Coroner's Court at the time due to ill health.^3^

Wakley was a social reformer, founding editor of *The Lancet,* a radical Member of Parliament and, crucially, a celebrated coroner who conducted over 20,000 cases. The story, from the aconite poisoning to the verdict by an associate of Ernest Hart, naturally gave rise to some gossip and there was a rumour, in later years, in the *British Medical Journal* (*BMJ*) office that her death was due to her husband's negligence, incompetence or possibly criminality. There was even talk, years later, that Hart had to flee the country after murdering his wife. However, this was clearly untrue as Hart did not have an extended visit abroad until 1869.

In 1872 Hart married his second wife, Alice Marion Rowland, (1848–1931), the sister of social reformer and educationist Henrietta Barnett. Rowland had herself studied medicine in London and Paris and was no less interested than her husband in philanthropic reform. The two sisters taught at Toynbee Hall, a charitable institution that works to address the causes and impacts of poverty in the East End of London and elsewhere. She was most active in her encouragement of Irish cottage industries and was the founder of the Donegal Industrial Fund and the Kells Embroidery schools.

## Journalism

Hart had been interested in writing for some time. As a young man he wrote articles for *Fraser's Magazine*, a literary journal whose contributors included Samuel Coleridge, William Makepeace Thackeray and John Stuart Mill. Here Hart argued the cause of emancipation of the Jews in Britain, as Jewish rights were still constricted by the Test Acts which were not repealed until 1872.

His journalistic career really began to take off in 1858 when he joined the staff of *The Lancet*. His duties included writing leading articles to order, proofreading and running the medico-parliamentary column. In 1865–1866, he participated in two important *Lancet* inquiries: into the cholera outbreak at Theydon Bois in Essex and into London's workhouse infirmaries, the findings of which contributed to the passage of the *Metropolitan Asylums Act*. In 1866, Hart's contract with *The Lancet* was abruptly terminated as the result of a quarrel with the editor, James Wakley, who resisted Hart's demand to be made joint editor. Later that year, the council of the British Medical Association (BMA) appointed Hart editor of the *BMJ*. He took up the post in January 1867 at an initial salary of £250 per annum. In making this appointment, it was later suggested, the BMA ‘had caught the editorial *leprechaun*, actually bought up the life and soul of the opposition establishment’.^3^ Although Hart remained editor of the *BMJ* at the time of his death, his tenure of office was interrupted for about a year during 1869–1870. The reason for his departure, while not entirely clear, was rumoured to have been connected to Hart's policy of making a steep increase in payments to *BMJ* contributors.^3^ Hart may have benefitted financially as a contributor to the *BMJ,* but his speedy return as editor is likely confirmation that no excesses had occurred. There is no definite evidence of how Hart spent this period, but he was unanimously reappointed in August 1870 after the *BMJ*'s undistinguished interregnum.

An early issue for Hart at the *BMJ* was the harsh criticism he meted out in editorials to the prominent gynaecologist Isaac Baker Brown which was to end Brown's career. Markham, Hart's predecessor, had already signalled his opposition to Brown's advocacy of clitoridectomies, as cures for epilepsy and hysteria, and Hart continued this policy, in no less than 10 editorials in the next year. Brown's career ended when he was accused of performing these procedures without consent of the patients and he was subsequently expelled from the Obstetrical Society of London.^4–6^ Other voices suggest that Brown was unfairly treated as the belief that female genitalia was responsible for hysteria was widely held at the time.^7^

Hart's achievement as editor of the *BMJ* was to transform it from a comparatively modest, obscure, low circulation and impecunious medical weekly, extending to no more than 20 pages, into a 64 page, highly respected and mass circulation journal. This success led to the national and international prominence of the BMA as a professional body. While writing for the BMJ he also regularly contributed to other journals such as the *Nineteenth Century*, the *Century* and the *Forum*. Hart was an adviser on medical literature to the publisher Smith & Elder, and he edited two of the firm's weekly journals, the *London Medical Record* (1873–1887) and the *Sanitary Record* (1874–1887). Under the title of *The Eternal Gullible*, Hart published a series of articles exposing the ‘shams’ of hypnotism and mesmerism. Besides his contributions to the journals he edited, he wrote numerous articles, pamphlets, lectures and reports on medical and other subjects. Some of his writings in the *BMJ, on topics such as vaccination, smoke abatement and professional ethics* were published in book form.

## Personality

As an individual, Hart was described as a ‘small slight figure, always with quick nervous movements of his body; a noble head and brow, pale, clear-cut face and large grey eyes that blazed out thoughts before they were spoken by the mobile lips and bell-like voice. His work was always done with the warmth of a sensitive and eagerly human mind.’^8^ However, given the difficult and sensitive issues, he confronted Hart was at once a controversial figure. His biography in the Dictionary of National Biography, written by P W J Bartrip, author of the history of the BMJ, *Mirror of Medicine*, described him as having ‘a forceful personality, being ambitious, opinionated, egotistical, self-confident, and inclined to intolerance.’^9^ At his memorial service his brother-in-law, Canon Samuel Barnett, said that he had a ‘strong critical power which dissected and ridiculed worn-out traditions’ and that ‘he made some enemies and mistakes… because he conceived that his first duty was not to his class but to the public.’^8^

As a young medical student, he was popular with his peers and after qualifying with the surgical staff.^8^ Sir John Simeon MP, secretary of the Metropolitan Workhouse Infirmaries Reform Association paid tribute to Hart's ‘tact, earnestness, business power and singular suggestiveness’.^10, 11^

**Figure 1.**
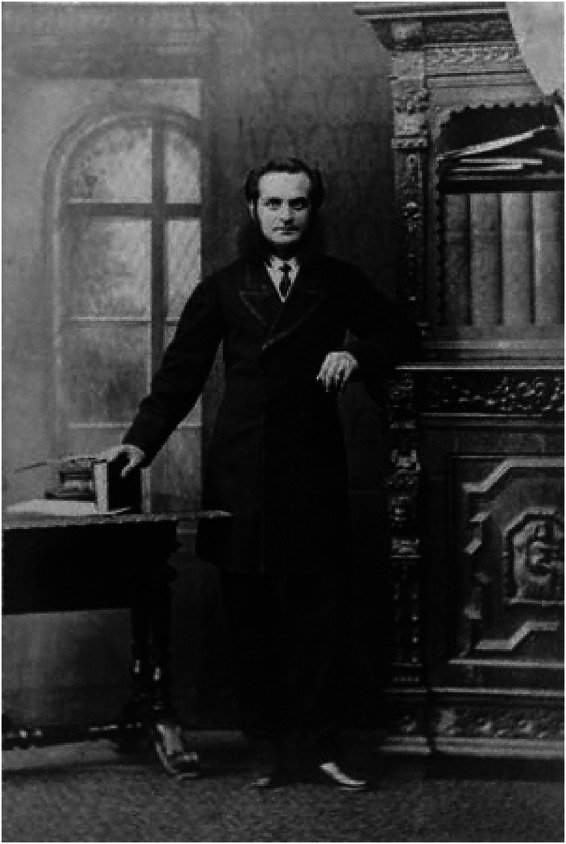
Ernest Abraham Hart (1835–1898) by Camille Silvy, 1866.

He was credited with his grasp of ‘the whole picture’ rather than having the ability to focus on small details, leading to his success in political campaigning at the detriment of any scientific research.^12^ A late tribute came in 1893 when the University of Durham conferred on him the degree of DCL (honoris causa) (Figure 1).

**Figure:**
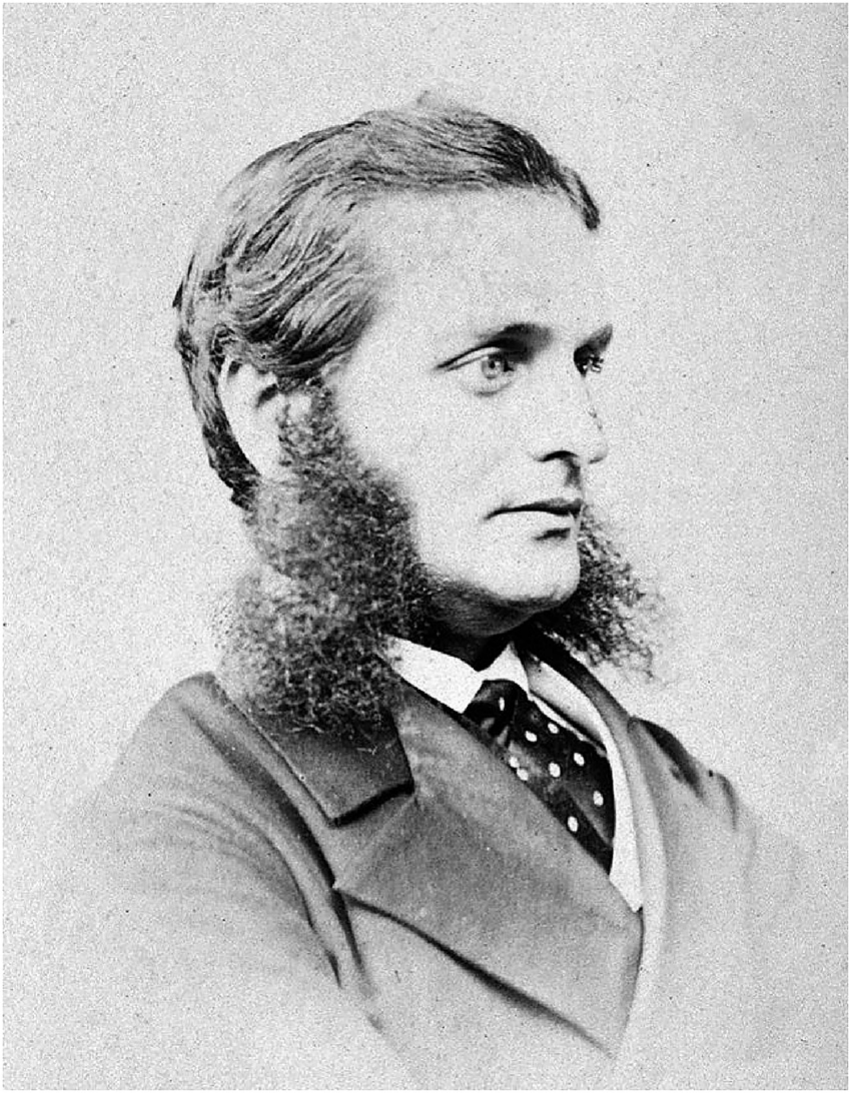
Ernest Abraham Hart

While many admired his intellectual, organisational and other abilities, he gained numerous enemies, including within the BMA. On several occasions, attempts were made to deprive him of his *BMJ* editorship, notwithstanding his acknowledged editorial achievements. Bartrip, noted that at least in part, the antipathy towards him was antisemitic, for Hart was staunch and outspoken in his Jewish faith.^3^ Bartrip also quotes Charles Adams:

To have pushed such a paper [the *BMJ*] into such a position is a feat of which any press-man might be proud, a feat which would hardly have been within the compass of any who had not in his veins the blood of that pre-eminently pushing race, his connection with which he would seem to be so curiously anxious to ignore.^3,13^

However, Hart was proud to be a Jew and his writings and community affiliations reflect this. At the age of 18 years, he published articles urging Jewish emancipation and his book *The Mosaic Code* (1887) gave a detailed account of Pentateuchal sanitation. He was frequently sought as a speaker at Jewish events and addressing a dinner of the Maccabeans in October 1892, he emphasised the importance of his Jewish background and referred to how he couldn’t, as a Jew, have undertaken medical studies at Cambridge University; yet he had been the first Jew in Britain to receive a university scholarship. Many Jews had followed him into medicine, and he described himself as belonging to ‘a quite prehistoric period’ though he was only 59 years old.^14^

## Social reform

**Figure 2.**
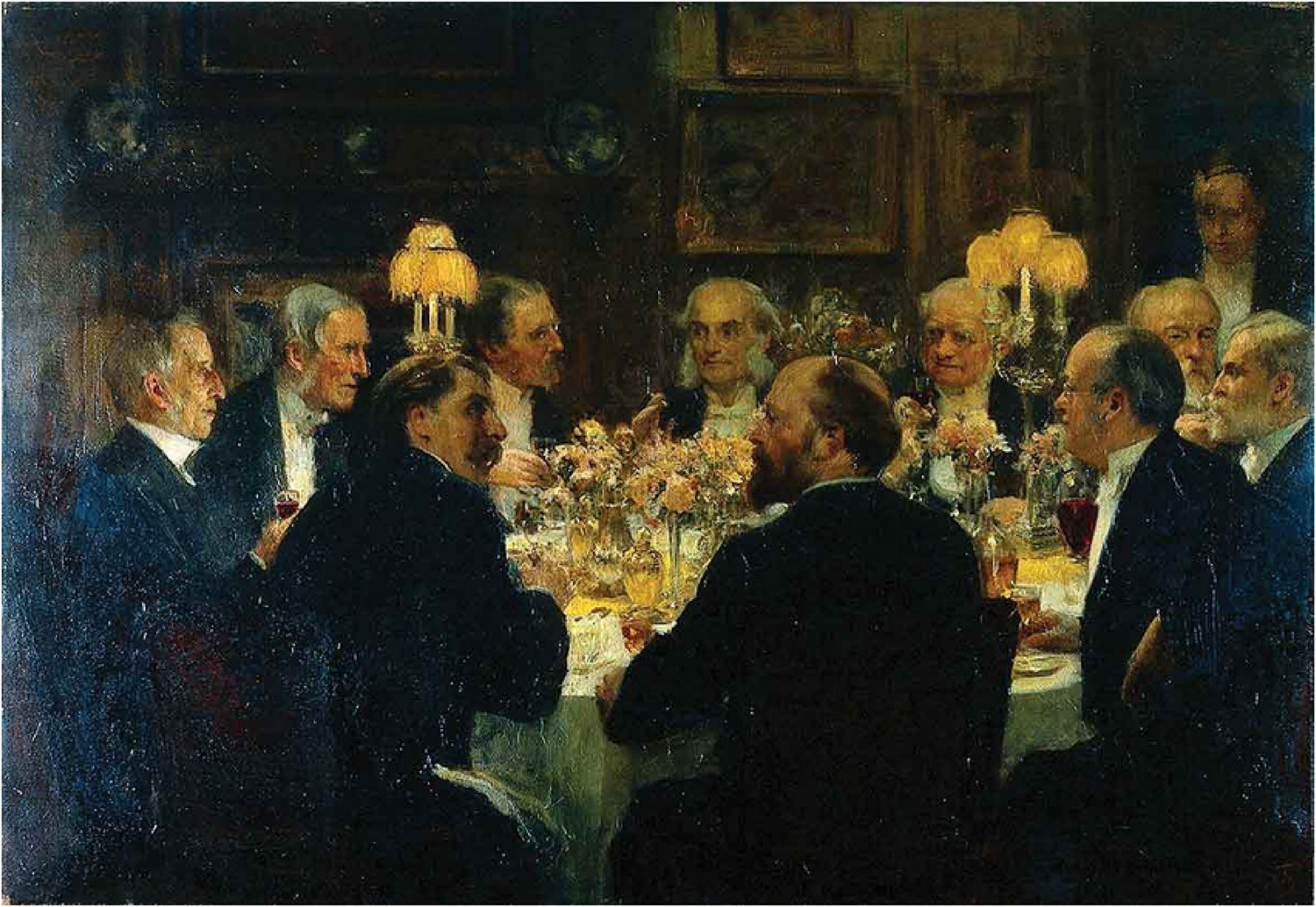
Solomon Joseph Solomon, An Octave for Mr Ernest Hart at Sir Henry Thompson's House, c. 1897, oil on canvas, 71.5 × 103.5 cm. Wellcome Library, London. Clockwise: Ernest Abraham Hart, Thomas Spencer Wells, Joseph Fayrer, Thomas Lauder Brunton, W.H. Broadbent, George Anderson Critchett, Victor Horsley, Richard Quain, James Paget, Sir Henry Thompson.

Hart was especially interested in social reform. He was a member of a commission in 1866 investigating the Poor Law infirmaries in London and the nursing of sick paupers. The commission report led to considerable improvements in the medical treatment of London's poor and led to the formation of the Metropolitan Asylums Board. In 1866, was appointed Editor of the *BMJ* to succeed Dr William O Markham who had been an editor for 5 years (Figure 2).

The editorship of the *BMJ* was a part-time position so from 1872 to 1897, he was Chairman of the Parliamentary Bills Committee of the BMA, which dealt with medical and sanitation measures, which were important in the era of cholera. This led to Public Health Acts for both London and for Scotland. He supported reforms in the appointment and careers of Medical Officers of Health and led many other health care initiatives. He was a consistent supporter of vaccination and his book *The Truth About Vaccination* refuted the arguments made by anti-vaccinators showing how the vaccinated were able to resist an attack of smallpox.

This, and his period of editorship of *The Sanitary Record* and being chairman of the National Health Society, provided the platform for him to promote several significant health reforms and counter what he saw as society evils that impacted on health. He helped to draw up the Infant Life Protection Act, directed against the lucrative practice of baby farming and the evils of the Barrack Schools where it was debated whether poor children even needed to be literate.^14^ Unwanted children under the age of 6 years had to be ‘disposed of’ and would be sent out of London to a ‘baby farm’ where the sum of two shillings could be expended on their care each week. Dickens took up the story of the baby farms in *Oliver Twist* where he described children lying on the floor, uncared for while the woman in charge kept the food expenditure down to seven pence ha’penny a week and pocketed the balance. The record of his public work covers nearly the whole field of sanitary legislation during the last 30 years of his life.

Hart had a hand in the amendments of the acts relating to medicine and public health, always promoting the medical profession above others in the public health field. He dealt with measures relating to notification of infectious disease, to vaccination and to the registration of plumbers; the improvement of factory legislation; remedying the legitimate grievances of Army and Navy medical officers and in the removal of abuses and deficiencies in crowded barrack schools.

Hart was an executive member of several bodies related to health care. One of these was the National Health Society which had been established in 1871 through the initiative of Elizabeth Blackwell and he was its chairman from 1877 to 1896. It promoted contemporary knowledge about public health through a variety of topical lectures designed to improve public health. It was also active in the promotion of formal training for women through courses in house health, childcare, nursing and ambulance work. In 1900, a couple of years after Hart's death, the National Health Society introduced the National Health Society Diploma which allowed women to work as health visitors, a service that was designed specifically to benefit the poor.

In 1881, he organised the Smoke Prevention Exhibition at South Kensington and, in the following year, was concerned about the damage by industrial smoke to health and property, the Smoke Abatement Movement. This, of course, was related to expanding Victorian manufacturing which relied heavily on coal and was not at all connected to the use of tobacco products, which Hart enjoyed and whose health consequences were not identified until several decades after his death. He also attempted to counter the adverse effects of excess alcohol in society by establishing popular coffee houses known as Coffee Taverns and he served the Coffee Taverns Company as its director.

Hart was also concerned about the illnesses being spread by infection in milk.^15–18^ In 1873, Hart, his family and several medical colleagues, including Charles Murchison and Sir William Jenner, had suffered from a particularly severe outbreak of typhoid which was perhaps the most well-known milk-borne disease in the Victorian era. At the International Medical Congress in London in 1881, when he was President of the BMA, Hart outlined his epidemiological research over the previous decade which linked typhoid fever, scarlet fever and diphtheria to infected milk. He wrote in 1881 that Radcliffe's 1873 Marylebone investigation

made so clear that the then sceptics became perforce converted to a belief in milk infection. The demonstration of this infection has indeed, by this time become so complete in this country, that its acceptance has now become practically universal.

In 1883, knowing doctors who were affected by chronic illness, he considered the possibility of setting up a medical self-help society.^19^ From this, the Medical Sickness Annuity and Life Assurance Society emerged, run voluntarily by doctors with Hart as its first chairman. Politically he was a liberal and he stood, unsuccessfully, as a Liberal parliamentary candidate in 1885. During the campaign, he had been questioned about his religion and he replied:

I believe in the One great living God. I was born a Jew, I am living as a Jew, and I shall die as a member of the great and glorious House of Israel.^12^

The Poor Law Committee, on which Henrietta Barnett and Ernest Hart served, reported in 1896 and unanimously condemned the barrack schools both on account of the dangers of disease, especially ophthalmia and skin infections, where so many children were crowded together, and on account of the emotional results of isolating children from the community and depriving them of individual care and affection. The alternatives of Village Communities, Scattered Homes and Boarding Out with Families were all recommended as preferable. The Committee criticised the organisation by the separate Boards of Guardians and unanimously recommended that a central authority should be given to the organisation of all pauper children. The majority of the Committee wanted this central authority to be drawn from the Boards of Guardians but Henrietta and Sir John Gorst recommended that the new authority should be the Education Department.

He was invited to attend the first meeting of the Indian Medical Association in Calcutta in 1894.^20^ There, Hart attacked the sanitary policies of the Indian Government clearly outlining their shortcomings which allowed the spread of cholera. His outspokenness was criticised at first, but his message was gradually accepted by the Indian government and helped to reduce the prevalence of cholera. He gave a public address in Hyderabad during a visit to India where he spoke openly about the risks of disease on what he called the ‘Pilgrim Path.’ The Path also included the Haj to Mecca from Muslim centres in India where cholera was also prevalent.

## Publications

At the time of his death, Hart was engaged in editing *Masters of Medicine*, a series of lives of eminent medical men but had completed the editing of only the first three volumes. The book on John Hunter was written by Stephen Paget, a surgeon and pro-vivisection campaigner, and on William Harvey by Sir D’Arcy Power, a surgeon and medical historian. The first volumes were published to much critical acclaim in Britain and America. A contemporary book review in the *Journal of the American Medical Association* commented:

The first two of these volumes are before us, and although much has been written of John Hunter and William Harvey, yet we must say that these volumes add very much to the general stock of knowledge concerning them… These lives are destined to become English classics.^21^

The third volume in the series, *Sir James Young Simpson and Chloroform* (1811–1870)

by H. Laing Gordon appeared in January 1897. Hart's other choices for the remainder of the series was Edward Jenner, Hermann von Helmholtz, William Stokes, Claude Bernard, Sir Benjamin Brodie, Thomas Sydenham and Andreas Vesalius.

Despite increasing health problems in later years, Hart undertook extensive travelling holidays, visiting the Far East, and North America where he was connected with the formation of many branches of the BMAs – one in Montreal was especially active. Dr Robert Warren, of the American Medical Association, said that his Association badly needed a man of Hart's calibre who ‘by his great push and enterprise had caused the *BMJ* to outrank any other journal published by any association’.

Hart was a heavy smoker and around 1890, he was diagnosed with diabetes. In 1897, his right foot was amputated as a result of herpetic spots, which were ulcerating and turning gangrenous. In spite of that, he carried on as editor of the *BMJ* until his death the next year even finding time to produce an expanded version of his 1881 account, listing all of the milk-borne epidemiological studies from the 1870s to the late 1890s.^8^ Hart died in Hove, Sussex, on 7 January 1898.

### Dr Phillips: A Maida Vale Idyll

Julia Frankau's book, *Dr Phillips: a Maida Vale Idyll*, using the pen name of Frank Danby, was published in 1887. In this, her first novel, Frankau painted an unflattering portrayal of *nouveau riche* London Jews and Jewish life which immediately attracted controversy. Many of the characters in the book seemed to be literary caricatures of figures, well known in Maida Vale, thus increasing the suspicion that the fictional Dr Phillips was based on the real-life Dr Hart. Both were successful physicians with an interest in medical journalism. Further, its depiction of Dr Phillips’ murder of his wife brought back vivid memories of the death of Hart's first wife. It was even claimed that Hart had added to the rumours by buying up every copy he could find and destroying them.^22^

Julia Frankau (1859–1916) became a successful novelist writing in a variety of genres. Her novel was republished in 1989 in an illustrated limited edition by the Keynes Press of the BMA with a lengthy introduction by Stephen Lock then Editor-in-Chief of the *BMJ*. Clearly, Lock was fascinated by the life of his famous predecessor, describing him as ‘one of the great medical editors of all time’.^1^ However, a century after Frankau's novel he noted the similarities and differences between Hart and Phillips finally concluding that the book stands on its own merits and that if ‘the one [Hart's prominence as a medical editor] draws attention to the other then we should all be grateful’.^1^

## Apollinaris

In 1872 Ernest Hart dined with George Smith, a partner in the publishing firm Smith, Elder & Co. and later founder of the *Dictionary of National Biography* and recommended Apollinaris, the German naturally sparkling mineral water, now owned by Coca-Cola, to Smith. The spring had been discovered by chance a couple of decades earlier in Bad Neuenahr, and the water was named after St Apollinaris of Ravenna, a patron saint of wine and soon became the leading natural table water in the world. Edward Steinkopff, Smith's business partner, formed a subsidiary English company to sell the water in Britain and Hart became its Scientific Adviser but did not take any stake in the company's stock.^23^

## Connoisseur of art

Hart's enthusiasms extended to hobbies such as growing roses in his country home at Totteridge, playing chess and dog and pigeon breeding. However, his main passion became Japanese art and his collecting began when he contacted Tadamasa Hayashi (1853–1906), the Japanese art dealer who introduced traditional Japanese art to Europe. From around 1882, Hart became a prominent collector of Japanese art and later joined the Japan Society, frequently giving lectures and writing on the subject. He enjoyed a worldwide reputation for his appreciation of Japanese art and many of his English essays were translated into Japanese. He had one of the finest collections of such objects, such as lacquerware, in Europe. In 1891 he travelled to Japan with his second wife, Alice Hart, where he received many honours for being permitted access to collections usually available only to royalty.

He was a founding member of the Council of the Japan Society, London in 1891. The interest of doctors in Japanese art was not a new phenomenon and two of Hart's contemporaries were, with him, the most prominent British collectors of Japanese art – objects, drawings and paintings. Alice accompanied her husband on his visit to Japan and also became a member of the Japan Society. Alice Hart was a noted watercolourist and, during her time as a member of the Japan Society, she read two papers in 1892 and 1900, the first female member to do so.

## Cremation

The BMA first discussed cremation at Cambridge in 1880, the year that the first crematorium, at Woking was completed. Hart was involved with the campaign for cremation from the outset. In an article on the topic 11 years later, Sir Spencer Wells wrote of the struggles to establish cremation legally which meant that the Woking crematorium could not begin functioning for 6years, until 1886.^24^ There was opposition from Parliament, medico-legal bodies, religious bodies and general public disquiet described as ‘widespread and powerful sentiment.’ Medico-legal requirements were clearly essential, but the discussion also revealed deficiencies in the current system of recording death with many internments being done without any certification.

More than a dozen medical men added their comments following those of Sir Spencer Wells. These included the genitourinary surgeon Sir Henry Thompson (1820–1904) a long-time friend of Hart. Hart described how cremation had been dealt with in a Shinto ceremony he had seen in Japan. He felt that no other system of burial could be ‘more decent or respectful.’^24, 25^

## Women in medicine

Hart took an active and very public role in supporting the movement of women into medicine. This was not easy for members of the BMA regarded the BMA as a male body and were resistant to the idea of women in the profession. His second wife Alice Rowland had studied medicine in Paris and later at the London School of Medicine for Women (LSMW). Hart became one of the LSMW governors when it was established in 1874. He and his wife also supplied money for two scholarships for female medical students.

## Obituary

There can be few who have merited an obituary in a scientific journal stretching to 11 pages.^8^ Yet, this unusual honour was given to Ernest Hart by the *BMJ* in its issue on 1 January 1898. One of the tributes in the *BMJ* was paid by Dr Elizabeth Garrett Anderson, Dean of the LSMW who referred to his role in promoting medical education for women and his long-standing support for them behind the scenes. An obituary in the *Journal of the American Medical Association* described how Hart's death had come as a shock to his many friends in America, even though his poor health was well known. The obituary noted that:

His death leaves a gap in the ranks of medical journalism which can never be filled. He had an instinctive delicacy of judgment, made few mistakes and popularized the *Journal* as no man preceding him had been able to do, and at the same time made it one of the financial successes of the age. He had learned the art of being aggressive without offending, and the *Journal* under his management has been right in its advocacy of all those reforms in which the whole profession is interested.^26^

The obituary concluded with a pithy account of Hart's character which summed up the various aspects of his character in a positive and accurate fashion:

As a writer he was forceful, accurate and aggressive. As a man, he was unassuming, polite and agreeable. As a physician he was well informed and, in certain lines, in advance of his time. He will be greatly missed for his place in medical literature was peculiarly his own.

A memorial service was held shortly after his death in Marylebone Parish Church, where his brother-in-law, Canon Samuel Barnett, conducted the service and his body was then transferred to Woking for cremation. Following cremation, his ashes were interred in the Willesden Jewish cemetery in north London.

## Legacy

Hart had once said that he ‘hoped to leave the world a little better than he found it’. His activities and successes were legions but his final legacy for the BMA was the establishment of a library and reading room, presenting many books and some valuable rare editions, including original works by Andreas Vesalius and the Hunter brothers.
